# Rifampicin-isoniazid induced fatal fulminant hepatitis during treatment of latent tuberculosis: A case report and literature review

**DOI:** 10.4103/0972-5229.68226

**Published:** 2010

**Authors:** Fahmi Yousef Khan, Fatima Rasoul

**Affiliations:** **From:** Hamad General Hospital, Department of Medicine, P.O.Box : 3050, Doha - Qatar

**Keywords:** Acute fulminant hepatitis, latent tuberculosis infection, antituberculosis treatment, hepatotoxicity

## Abstract

A 42-year-old Indian man received 450 mg rifampicin (RIF) and 150 mg isoniazid (INH) daily after being diagnosed of a latent tuberculosis infection. Baseline serum aminotransferase and total bilirubin levels were within normal limits. On day 31 of treatment, the patient experienced epigastric discomfort and general malaise and one week later he developed nausea and episodic vomiting. The patient missed his first scheduled clinic appointment and he continued taking RIF-INH despite his symptoms. He visited the tuberculosis clinic on day 47 of treatment where he was found to be jaundiced and his liver enzymes were elevated. RIF-INH was stopped and the patient was admitted to our hospital as a case of RIF-INH induced hepatitis. On the 7th day of hospitalization, the patient developed consciousness disturbance with flapping tremor and high ammonia level. The patient was diagnosed with fulminant hepatic failure and transferred immediately to the medical intensive care unit, where he died 4 days later.

## Introduction

Tuberculosis (TB) remains a common health problem in developing countries. In Qatar, it has an incidence rate of 60/100,000, and an average of 491 cases are diagnosed annually.[1] The disease mostly affects expatriates. Majority of cases of active tuberculosis stem from patients with latent tuberculosis infection (LTBI) (patient with a positive tuberculin skin test but no evidence of active disease). Preventing active tuberculosis by treating LTBI is a major element of the national strategy for eliminating tuberculosis in Qatar. In 2008, a total of 1142 cases of LTBI were treated at the TB clinic in the state of Qatar. A 4-month course of treatment with Rifampicin-Isoniazid (RIF-INH) for LTBI is one the regimens that being widely used in this country. This usually well-tolerated regimen occasionally can result in severe adverse effects including hepatitis and hepatic failure. Herein, we present the case of a 42-year-old Indian man who died of fulminant hepatic failure in the course of receiving four months of treatment with RIF-INH for his latent tuberculosis. To the best of our knowledge, this is the first reported case of RIF-INH induced fatal fulminant hepatitis in Qatar, since the introduction of this regimen to this country. Our case highlights the importance of patient’s education and clinical monitoring during the treatment of LTBI to recognize hepatotoxicity in time and to prevent progression to acute liver failure.

## Case Report

A 42-year-old Indian man was admitted to our hospital, through the TB clinic, because of antituberculosis drug-induced hepatitis. 47 days before admission, he had been diagnosed as having LTBI. He was therefore placed on 450 mg RIF and 150 mg INH daily (starting on 11 February 2009). Baseline serum aminotransferase and total bilirubin levels were within normal limits. He was instructed to stop taking RIF-INH if he developed symptoms suggestive of hepatitis (abdominal pain, emesis, jaundice). Moreover, the patient was scheduled monthly TB clinic visit for clinical assessment and monitoring for signs and symptoms of possible RIF-INH adverse effects. Past medical history of the patient was unremarkable. The patient was a healthy and active man who did not consume alcohol and had no known chronic illness such as liver disease. He had no history of drug abuse or blood transfusions, and no history of recent illnesses or contact with sick people.

On day 31 of the treatment, the patient experienced epigastric discomfort and general malaise and one week later he developed nausea and episodic vomiting. The patient missed his first scheduled clinic appointment and he continued taking RIF-INH despite his symptoms. On day 38 of treatment, his colleagues observed yellowish discoloration of his sclera and advised him to visit the clinic. His general condition worsened and he came to the TB clinic on day 47 of treatment. Liver function test, blood chemistry and CBC were performed on the same day. Alanine aminotransferase (ALT) was 1,192 U/L, aspartate aminotransferase (AST) 1,597 U/L, total bilirubin 237μmol/L and INR 1.4 [Table 1]. RIF-INH was stopped and the patient was referred to our hospital for admission as a case of RIF-INH induced hepatitis. On examination upon arrival to the hospital, he was jaundiced, very anxious, and hyperventilating but there was no flapping tremor. Abdominal examination showed epigastric tenderness without shifting dullness. Serological studies, including tests for hepatitis A, B, C, E viruses, cytomegalovirus, HIV, Epstein-Barr virus, herpes simplex viruses 1 and 2, gave negative results. Furthermore, markers related to autoimmune hepatitis were negative. α1-antitrypsin and ceruloplasmin were normal. Ultrasound results of the abdomen were normal.

**Table 1:** Patient's investigations during hospitalization

Investigation	Date of admission										
	25/01	28/03	31/03	1/04	3/04	6/04	7/04	8/04	9/04	10/04
ALT (U/L)	21	1216	1286	1401	1370	1062	1062	1170	1489	1207
AST (U/L)	25	1597	1870	1870	1669	1610	1610	1726	1726	1253
ALP (U/L)	75	134	113	105	95	77	78			114
T. bilirubin (μmol/L)	18	234	229	247	278	292	357	328	379	389
T. protein (g/L)	80	85	76	73	68	70	71	74	64	62
S. albumen (g/L)	36	36	29	28	25	21	30	28	23	21
INR	1.1	1.4	2.3	2.1	2.7	3.7	4.1	2.1	4.5	5.5
Ammonia (μmol/L)						119	110	117		
HB% (g/dL)	13.3	11.2	12.2	11.2	12.2	11.4	11.5	10.8	10.9	9.8
WBC (/μL)	9000	7500		6600	7200	5100	5300	4400	5900	5400
Platelets (×10^9^/L)	219	239		198	202	143	147	148	137	102

During his stay in the ward the patient received Vitamin K and fresh frozen plasma, metoclopromide, ranitidine and intravenous fluid with a hepatic diet. The patient developed fever on the third day of hospitalization. Full septic workups including blood and urine cultures were carried out. Blood film for malaria parasite was negative, and the patient was covered with ceftriaxone. On the 7 ^th^ day of hospitalization, the patient developed consciousness disturbance, clinical examination disclosed a drowsy jaundiced patient with flapping tremor. Serum ammonia was high, whereas, blood and urine cultures were negative. The patient was admitted to the medical intensive care unit as a case of fulminant hepatic failure evidenced by marked elevation of hepatocellular enzymes, prolonged prothrombin time, hyperbilirubinemia and hyperammonaemia [Table 1]. The patient was given Vitamin K and fresh frozen plasma to correct the prolonged INR, and lactulose. Broad-spectrum antibiotic were initiated. Despite all these measurements, liver failure progressed and a deep coma together with a marked tendency to bleed developed. The patient died on 11 April 2009, 2 months after the initial administration of RIF-INH.

## Discussion

In 2000, the Centers for Diseases Control (CDC) of the United States published a detailed set of guidelines on targeted tuberculin testing and treatment of LTBI.[2] Targeted tuberculin testing for LTBI is a strategic component of tuberculosis control that identifies persons at high risk for developing tuberculosis who would benefit by the treatment of LTBI, if detected. INH remains the drug of choice for treatment of LTBI.[2–4] It is the only drug used for LTBI that has been evaluated on a large scale in randomized controlled trials. Treatment of LTBI with INH greatly reduces the likelihood that active tuberculosis will develop and decreases the number of adults with active disease who will transmit infection to others, as demonstrated by a number of controlled clinical trials.[5–7] The effectiveness of this drug for the treatment of LTBI has been reported to range from 25% to 92%;[2,8] this wide range is attributed to differences in medication adherence.[9] The preferred duration of treatment with INH for LTBI in all patient populations is 9 months.[2,10] However, this regimen has been burdened with suboptimal rates of therapy completion and concerns about hepatotoxicity and infection with INH- resistant organisms. These problems have stimulated considerable interest in finding shorter and safer regimens for the treatment of LTBI. Therefore, other alternatives were recommended by the CDC, which include a 4-month RIF and a 2-month of daily rifampicin- pyrazinamide (RIF-PZA).[2] Treatment of LTBI with 4-months RIF leads to fewer serious adverse events and better adherence than 9 months of INH,[11,12] whereas subsequent use of the RIF-PZA regimen under routine conditions was associated with unacceptably high rates of hepatotoxicity.[13,14] As a result, revised guidelines that RIF-PZA should not be used to treat LTBI in either HIV-infected or uninfected persons were published by the American Thoracic Society and the CDC in 2003 and were endorsed by the Infectious Diseases Society of America.[15] A 3-month regimen of INH plus RIF is also recommended as an alternative for treatment of LTBI by the British Thoracic Society.[16] The available evidence suggests that 3 to 4-month therapy with RIF-INH is equivalent to standard therapy with INH in terms of efficacy, the proportion of severe side effects that occurred, and mortality.[16,18] Moreover, patient adherence with 3 to 4-month RIF-INH is excellent when compared to 9-months INH.

Drug-induced liver disease is a well-known side effect of several drugs that are used for the treatment of active tuberculosis or latent tuberculosis infection. A meta-analysis has shown an incidence rate of liver toxicity of 2.6% with RIF and INH co-administration, but only 1.1% with RIF alone, and 1.6% with isoniazid alone.[19] Hepatotoxicity can range from asymptomatic elevation of serum transferases to hepatic failure requiring liver transplantation.[20] Deaths due to RIF-INH induced fulminant hepatitis have been reported, albeit rare in occurrence. Hepatotoxicity is reported mainly in patients with risk factors such as advanced age, female sex, pre-existing liver disease, alcohol consumption, slow acetylation status, concomitant use of hepatotoxic drugs, and a high dosage of antituberculosis medications in relation to body weight.[21–25] The factor of greatest clinical importance for the development of severe hepatotoxicity is probably continuation of the treatment once hepatic dysfunction has initiated.[26]

It is likely that our patient suffered acute hepatic necrosis as a result of RIF-INH ingestion; there was no serological evidence of infection with hepatitis A, B, C, or E, cytomegalovirus and Epstein-Barr virus. Markers to autoimmune hepatitis were negative. Furthermore the patient had no obvious risk factor for hepatotoxicity prior to RIF-INH therapy; he was asymptomatic and he did not consume alcohol and had no known chronic illness such as liver disease. He had no history of drug abuse. His baseline liver function measurements were within normal limits. But unfortunately, he missed his scheduled clinic appointment; moreover, he did not seek medical help early and continued taking his medications while symptoms were developing. This is possibly the exacerbating factor for liver failure in our patient, which has been noted in other reports of severe liver injury.[27]

Because our patient was taking a combination of anti-TB drugs, it is difficult to conclude which drug was the main culprit for hepatitis. It is noteworthy that RIF has been implicated in increasing the likelihood of liver injury when used in combination with INH or other antituberculous agents.[19]

The pathogenesis of RIF-INH induced hepatotoxicity is not entirely clear, but the proposed mechanisms may include dose-related toxicity,[28] oxidative stress,[29] lipid peroxidation,[30] immune-related,[31–33] induction of liver enzyme in the hydrolase system, thus enhancing the toxicity of some of the INH toxic metabolites,[34] activation of CYP2E1,[35] reduced glutathione level,[36] glutathione S-transferase M1,[37] histocompatibility Complex Class II associated HLA-DQ alleles,[38] choline deficiency leading to lowering of phospholipids protein synthesis with alteration in cell wall configuration[39] and hypersensitivity to RIF-INH.[40]

Once severe organ toxicity initiates, the survival rate remains under 10% if organ transplant is not available. Factors associated with high mortality in patients with drug-induced hepatotoxicity include: hepatic encephalopathy, ascites, jaundice, alcohol abuse and high direct bilirubin level.[41] All these factors except ascites and alcohol abuse were present in our patient.

In summary, efforts to eradicate tuberculosis by treating LTBI will expose a greater number of patients to the risk of potentially serious hepatotoxic effects of antituberculosis drugs, since the choice of effective safe alternative antituberculosis drugs is limited. Therefore, the factor of greatest clinical importance in the treatment of LTBI is probably early recognition of hepatic dysfunction, which is possible only by regular monitoring and education of the patient. More importantly, at each visit patient education should be reinforced, including giving the patient a written summary of possible adverse effects and what to do in the event that a possible adverse event occurs, since many patients, including our patient, who ultimately die or require liver transplants frequently, have a history of continued use of these medicines even after symptoms of hepatotoxicity, including jaundice, have appeared.
